# Anti-inflammatories for depression: challenges and ASPIRations

**DOI:** 10.1016/S2215-0366(25)00308-6

**Published:** 2025-10-17

**Authors:** Courtney Worrell, Bernhard T. Baune, Francesco Benedetti, Annamaria Cattaneo, Livia De Picker, Jennifer C. Felger, Stefan M. Gold, Femke Lamers, Marion Leboyer, Giulia Lombardo, Fanni-Laura Mäntylä, Andrew H. Miller, Valeria Mondelli, Brenda W.J.H. Penninx, Luca Sforzini, Dan J. Stein, Benedetta Vai, Erik Van der Eycken, Anthony Woods, ASPIRE Study Consortium, Carmine M. Pariante

**Affiliations:** 1 Department of Psychological Medicine, Institute of Psychiatry, Psychology, and Neuroscience, King’s College London, London, UK; 2 Department of Psychiatry, University of Münster, Münster, Germany; 3 Division of Neuroscience, Psychiatry & Clinical Psychobiology Unit, IRCCS Ospedale San Raffaele, Milano, Italy; 4 Biological Psychiatric Unit, IRCCS Istituto Centro San Giovanni di Dio Fatebenefratelli, Brescia, Italy; 5 Department of Pharmacological and Biomolecular Sciences, University of Milan, Milan, Italy; 6 Collaborative Antwerp Psychiatric Research Institute, Faculty of Health Sciences, University of Antwerp, Antwerp, Belgium; 7 Department of Psychiatry and Behavioural Sciences, Emory University School of Medicine, Atlanta, GA, USA; 8 Department of Psychiatry, Campus Benjamin Franklin, Charité – Universitätsmedizin Berlin, Berlin, Germany; 9 Department of Psychiatry, Amsterdam Public Health and Amsterdam Neuroscience, Amsterdam UMC, Vrije Universitiet, Amsterdam, the Netherlands; 10 FondaMental Foundation, Creteil, France; 11 Department of Addictology and Psychiatry, Université Paris-Est Créteil, AP-HP, Translational Neuropsychiatry laboratory, Inserm U955, PEPR PROPSY, Créteil, France; 12 Global Alliance of Mental Illness Advocacy Networks-Europe, Brussels, Belgium; 13 National Institute for Health and Care Research (NIHR) Maudsley Biomedical Research Centre at South London and Maudsley NHS Foundation Trust and King’s College London, London, UK; 14 Department of Psychiatry & Mental Health, University of Cape Town and Groote Schuur Hospital, Cape Town, Republic of South Africa

A recent article in *The Lancet Psychiatry* describes the problematic state of drug development in psychiatry, and identifies, among the obstacles, the lack of “science-based innovation”.^1^ Here, we propose that using anti-inflammatories in people with the so-called inflammatory subtype of depression is one such science-based innovation ripe with opportunity for success. We also discuss the challenges and unknowns, and map a way forward for anti-inflammatory drug development in depression.

A multitude of studies and meta-analyses have shown that, compared with people who have never experienced depression, people with major depression show, on average, higher concentrations of peripheral blood biomarkers of inflammation, such as the acute phase protein, C-reactive protein (CRP), and the proinflammatory cytokines, interleukin (IL)-6 and tumour necrosis factor (TNF)-alpha, as well as increased absolute counts and activation of immune cell subpopulations.^2^

In the background of this immune activation, there is also evidence of a unique subtype of inflammatory depression in individuals who have clinically significant, low-grade inflammation, defined by concentrations of CRP above 3 mg/L.^2^ Individuals with this inflammatory subtype have an overrepresentation of specific clinical features. Notably, they have more severe motivational anhedonia, that is, a deficit in the anticipation and willingness to work for potential rewards that is often accompanied by an increased sense of fatigue and lack of energy.^2,3^ This can be measured as reduced scores on dedicated scales (for example, the Multidimensional Fatigue Inventory or the anhedonia subscale from the Inventory of Depressive Symptomatology-Self Reported) and as a decreased functional magnetic resonance imaging (fMRI) task-based activation in the Dorsomedial Prefrontal Cortex and the striatum, brain areas that are important for reward processing and that are sensitive to increased inflammation.^2,3^ Additional clinical features of this inflammatory subtype include more severe sleep and appetite problems, metabolic abnormalities (obesity, dyslipidemia), a more chronic illness course, and a weak response to conventional antidepressants.^2^ Most importantly, this subtype may be more likely to respond clinically to anti-inflammatories.

However, recent clinical trials of anti-inflammatories in depression have shown conflicting and often negative findings, possibly because of the inconsistency of study designs in terms of study populations, anti-inflammatories tested and use of immune biomarkers.^4–7^ Some of these studies^4,5^ have been criticised because they did not enrich for patients with the inflammatory subtype.^8^ However, the studies that did attempt post-hoc stratification for immune biomarkers,^3,7,9,10^ even if at times underpowered, indicate that results vary with the biomarkers or the anti-inflammatories used, or with other clinical features of the study populations. For example, a CRP concentration above 3 mg/L predicts response to 4 weeks of minocycline^9^ and 12 weeks of infliximab, especially in symptoms related to reward,^3^ but does not predict response to 6 weeks of celecoxib^10^ or 12 weeks of simvastatin,^7^ assessed as changes in total depressive symptoms scores. This inconsistency runs the risk of killing this science-based innovation approach before we can fully comprehend it, and indeed, the stratified analyses conducted so far have highlighted the biological and clinical complexity underpinning the conflicting findings.

To this aim, we propose a range of questions that need to be addressed before we can decide whether the use of anti-inflammatories in depression should be implemented or abandoned. Are there immune biomarkers, or a combination of biomarkers, that reliably identify individuals with depression who will respond to anti-inflammatories? Are the biomarker(s) the same for different anti-inflammatories, or is the prediction specific for a given drug or pharmacological mechanism? Will prediction accuracy improve if we add other measures relevant to the inflammatory subtype, such as motivational anhedonia or metabolic abnormalities, to the immune biomarkers? Can a small number of such features, measurable in a clinical context, be sufficient to capture all the variability in response prediction? Are there other, more research-intensive biological measures, such as transcriptomic profiles or neuroimaging changes, that can improve this response prediction? Which clinical scales are best suited to capture the specific symptom changes in clinical trials with anti-inflammatories for depression?

Of course, science-based innovation will not work if it is not embraced by the people who might benefit from it. Hence, we also want to know the views of people with depression on the broader role of inflammation in mental health, and on the acceptability and perceived benefit of anti-inflammatories for depression. And also, how do we present such a concept to the general public, and how will it affect mental health literacy and stigma?

We aim to answer all these questions in our Wellcome-funded ASPIRE study (Advanced Stratification of People with Depression Based on Inflammation), now starting its second year, bringing together immunopsychiatry research centres and associations of people with lived experience of depression from the UK, Europe, the USA, and South Africa. (For more on ASPIRE, including the opportunity to submit data, please write to us.) After dedicating the first year to harmonisation procedures and protocol development, we are now ready to combine all existing data from completed or ongoing clinical trials with anti-inflammatories for depression. We will first prepare individual-level meta-analyses of clinical data and new, harmonised measures of immune biomarkers. Then, we will develop a machine-learning-based predictive algorithm for response to anti-inflammatories in people with depression, and test its acceptability in different countries and socio-economic and cultural contexts.

With such an ambitious plan, we *aspire* to deliver personalised medicine in psychiatry.
